# Blood Money: Is it Time to Incentivize Blood Donation in India?

**DOI:** 10.7759/cureus.64692

**Published:** 2024-07-16

**Authors:** Satvik N Pai, Madhan Jeyaraman, Naveen Jeyaraman, Sankalp Yadav

**Affiliations:** 1 Orthopaedics, People's Education Society (PES) University Institute of Medical Sciences and Research, Bengaluru, IND; 2 Clinical Research Associate, Viriginia Tech India, Dr MGR Educational and Research Institute, Chennai, IND; 3 Orthopaedics, ACS Medical College and Hospital, Dr MGR Educational and Research Institute, Chennai, IND; 4 Medicine, Shri Madan Lal Khurana Chest Clinic, New Delhi, IND

**Keywords:** blood, blood bank, compensation, monetary incentive, blood donation

## Abstract

Blood donation, a fundamental act of altruism, has undergone significant evolution over the centuries. Despite medical progress, the persistent challenge lies in securing an adequate supply of blood and its derivatives. This article critically examines the proposal to introduce monetary incentives for blood donation, delving into ethical, legal, and pragmatic dimensions. India’s current framework staunchly upholds voluntary, unpaid donations. However, global practices diverge significantly. Notably, India previously permitted monetary compensation for blood donation but later prohibited it due to concerns about infection transmission. Monetary incentives offer a potential solution to address key deterrents faced by potential donors. Health anxieties, time constraints, and fear of infection often discourage voluntary donations. By providing financial motivation, we may enhance donor participation and bolster the reliability of the blood supply. While the benefits are evident, caution is essential. Implementing monetary incentives necessitates robust safeguards. Preventing the exploitation of vulnerable populations and ensuring the safety of blood products remain paramount. Striking this delicate balance requires careful consideration. By analyzing ethical, legal, and practical facets, we navigate the intricate landscape of blood supply management.

## Editorial

Blood donation has always been considered a noble deed, and rightfully so. It helps another human being in dire need. In some cases, it is life-saving. The first reported blood transfusion in a human was done by physician Jean-Baptiste Denis as long ago as 1667. Since then, the science and methodology of blood transfusion have seen transformative advancements. However, at its very core, the ethos behind blood donation and transfusion remains the same: that of a human being helping another by donating a part of themselves. As science and society have evolved, it is inevitable that blood transfusions will be influenced by those changing environments. Blood and blood products have always been a scarce resource, and acquiring sufficient blood products to meet requirements has always been a challenge in society. Unfortunately, there have been several instances all over the country of individuals having to pay with their lives when there has been a shortage of blood or a delay in acquiring it. A solution proposed to this problem has been providing monetary incentives for blood donation. In this article, we look at monetary incentives for blood donation ethically, legally, and, most importantly, practically. We critically analyze the different perspectives on monetary incentives for blood donation, the problems it would solve or create, and dissect what the solutions might be.

The current legal scenario in India and around the world

Blood donation in India is governed primarily by the Drugs and Cosmetics Act of 1940, where blood does not really fit into the regular description of a drug or cosmetic agent. But as per the law, human blood is covered under the definition of ‘drugs’ under Section 2(b) of the Drugs and Cosmetics Act, and whole human blood is included in the Indian Pharmacopoeia under Schedule ‘C’ of the Drugs and Cosmetics Rules [1]. Interestingly enough, that law did not outlaw monetary compensation for blood donations. As a result, monetary compensations were being given out to donors at various blood banks in the country. This changed in the 1990s, when, due to the increasing incidence of HIV at the time, the Supreme Court of India banned paid blood donations [2]. The government of India adopted the National Blood Policy in 2002 with the aim of developing a system that ensures an adequate and safe blood supply [1]. As per the current laws, all blood donations have to be voluntary and non-monetary. But it is not just in India; the World Health Organization recommends that all blood donations should come only from unpaid voluntary donors. Many countries, however, do not agree with this principle and have always allowed for a monetary incentive to ensure adequate blood donation and the availability of blood products for their citizens. A survey conducted in 2006 found that only 49 of the 124 countries surveyed had outlawed monetary payments for blood donation [3].

Reasons behind voluntary blood donation

The motive behind blood donations currently is believed to be the desire to help others, with the recipient being a known or unknown person for the donor. The effectiveness of this motivation is demonstrated by the fact that convincing individuals to donate blood has always been a challenge. Providing monetary incentives has therefore been suggested as a solution to this problem, which will motivate people to donate blood. But there are several reasons why blood donation has only been allowed when done voluntarily until now. The most significant reason given for this is that if monetary compensation is allowed for blood transfusion, it would open the gates for exploitation. It is argued that low-income individuals would then be incentivized to donate blood for the sole purpose of financial gain. While the financial benefit would be compensation, it could lead to several individuals compromising their health and safety for a few quick bucks [4]. It could also lead to the creation of a marketplace for blood and blood products. Since monetary payments would need to be shelled out to the donors, the blood banks would have to then increase the price of such products. This could make blood and blood products expensive and out of the reach of financially weaker sections of society. The safety of the blood donated has always been raised as a potential problem that would arise. It is postulated that certain groups of individuals, such as intravenous drug abusers and sex workers, would be frequent donors with the intention of monetary benefit. Since they are considered high-risk for blood-borne infectious diseases like HIV and hepatitis, it could raise concerns about the safety of the blood products. It might also bring into question the very voluntary nature of the donation. Citing the financial gain to be made, certain people or organizations may utilize their position of power over certain persons to coerce or pressure them into blood transfusion while the monetary benefit is usurped by another.

The ethics of monetary incentives for blood donation

Blood donation is currently seen as ethical beyond doubt because the sole purpose of blood donation is said to be altruistic. Altruism can be defined differently, but its basic meaning is a desire to selflessly help others. This is quite interesting when applied to blood donation. Firstly, we question whether blood donation is currently truly altruistic. A majority of blood donations in the country are direct donations for a specific recipient, i.e., blood donations done specifically when a family member, friend, or acquaintance is in need of a blood transfusion. Such an act cannot really be termed selfless, as it is clearly intertwined with the interpersonal relationship between the donor and recipient. Blood donations done without any specific donor in mind can be considered altruistic, but this brings us to our second question. Such an important medical resource requires relying on the altruistic behaviors of a population. Altruism is considered a very high moral principle, one that is scarce in society and dwindling further as materialism increases. Altruism is not relied upon for any other essential resource, as it would be considered a very vulnerable situation for society to be in. Why, then, are we agreeable to leave those requiring blood transfusions at the mercy of finding those altruistic few? We believe that an essential commodity like blood and blood products has to be sourced sufficiently and reliably to ensure that there are sufficient resources available at the time of need.

Now, if we were to provide monetary incentives for blood donation, would that be ethical? When we use the term 'incentive', we are inherently painting the act and motive in dim light. We suggest an alternative perspective. What if the financial remuneration given is instead seen as ‘compensation’ for the help provided? We cannot ignore the fact that whatever the motive of the blood donation, the blood donated will help the recipient. Would it really be unethical to reward the individuals who are coming forward to help? It must be noted that the mere offering of financial remuneration does not mean everyone would be willing to donate blood. Then, would it not be fair to provide some acknowledgment of this volunteering? Governments and agencies all over the world have recognized for several decades now that some form of acknowledgment or positive reinforcement is essential to ensure blood donors are encouraged to donate again and to motivate others to donate. Various forms of this non-monetary compensation have been tried with varying success. Providing a blood donor certificate, social recognition of blood donors, and blood donor T-shirts are all examples of non-monetary compensation. The caveat with non-monetary compensation is that its effectiveness depends on the suitability of the donor profile, which is highly variable and indigenous to a particular fraction of society. Here again, the one language that is universal in its appeal is monetary compensation. How much does giving money versus a gift change the ethics? Not very much, we think.

The third ethical debate is that of exploitation. There is no denying that providing a monetary incentive for blood donation would be more attractive to financially weaker sections of society than to those for whom the monetary compensation would be meager compared to their incomes. Would this then lead to a situation where the more fortunate sections of society benefit from the monetary needs of the weaker sections? Is that social injustice? We don’t think so. Social inequalities are present and will continue to be present in every society. That is an issue with a far greater scope and horizon in the economic fabric of a country or population for it to be significantly influenced by a small factor like blood donation alone. Providing monetary compensation does not significantly bridge the economic gap, nor does it widen it. Even if we are not looking at the grand scheme of things and focusing on the social injustice of blood donation specifically, we ask if it can even be constituted as an injustice if the donors are being compensated sufficiently. From the perspective of an individual who is struggling for economic survival, would he or she see this small avenue for monetary benefit as an opportunity or as exploitation? We think it is more likely the former. Nevertheless, if it is later, there is absolutely no compulsion on the individual to donate blood. Hence, it can be used only by those who see it as something beneficial to themselves and only when they want to. That, we think, eliminates the question of social opportunism or injustice.

Testing the practicality

As one might have figured, we do not see an ethical roadblock to monetary compensation for blood donation. So now let us delve deeper into the practicality of the proposal. First and foremost, let us recognize and state the problem. The problem, as it always has been, is motivating individuals to donate blood. But why is convincing someone to donate blood hard? First, there is the fear of consequences for the health of the donor due to volume loss. Many individuals believe that donating blood would make them vulnerable to health risks due to decreased blood volume. There is overwhelming scientific evidence that any implications for the health of the healthy donor due to the decreased blood volume are temporary. But this goes against intuitive thinking and, therefore, will require the education of the donor and creating awareness among society in general that blood donation is safe. Creating that awareness in itself is another challenge, though. Various modes of awareness creation have been employed, including social media campaigns. While many of them have succeeded in reaching small fractions of the population, a large proportion of the population of the country still has very poor awareness about blood donation. Second, time spent and inconvenience to the donor. Blood donation requires an individual to spend a minimum of half an hour for the blood donation process, oftentimes more if you consider the time required for prior blood testing and time spent traveling to the blood donation center. This can be reduced to some extent by organizing blood donation camps in various populous areas; however, the fact that it would still require individuals to sacrifice some part of their day for the activity is undeniable. Thirdly, there is the risk of acquiring a blood infection, especially HIV and hepatitis. These not only pose a health hazard but also come with a social stigma attached to them. Prior screening of all donors has drastically decreased the incidence of these, but rare lapses still do occur. Despite the solutions that are being undertaken for each of these three problems and the attempts at making blood donation as safe and convenient as possible, it starts to become obvious that convincing individuals to take on that risk and inconvenience to donate blood is always going to be challenging [5]. Relying on the altruistic solutions that are being undertaken for each of these three problems and the attempts at making blood donation as safe and convenient as possible, it starts to become obvious that convincing individuals to take on that risk and inconvenience to donate blood is always going to be challenging [5]. The ideology of individuals to trump these factors is not a reliable strategy. As the saying goes, you can lead a horse to water, but you can’t make it drink unless it wants to. Therein lies the paradigm-shifting ability of introducing monetary incentives for blood donation.

Now that we’ve also highlighted the need for monetary incentives, let us probe further into the practical problems once you introduce monetary incentives for blood donation. After all, if we’ve argued that there is a need for monetary incentives and it is not unethical, then what is stopping India and so many other countries from adopting this policy? The first concern is that it would encourage low-income individuals to neglect the health risks and donate for monetary benefit. This we do not actually think would happen. While it would be ideal to educate individuals to not put their own health in jeopardy, even if an individual attempts to, the blood donation system must be robust and efficient enough to not allow for such blood donation. Even in voluntary blood donations now, screening of the donor’s hemoglobin levels prior to blood donation must happen. This safety check would disqualify any individual who is excessively donating blood or is not fit to be donating blood. If an individual wants to repeatedly donate blood and has met the criteria for medical fitness to donate blood, he or she should be allowed to do so. Coming to the concern raised about the probable increase in blood-borne infections like HIV and hepatitis, this again should be something that the blood bank or blood collection center must ensure. The onus for ensuring the safety of blood collection, storage, and transfusion is on the blood bank facility and not on the donor or recipient. It is a universal procedure mandated by law that all donors must be screened for blood-borne infections like HIV and hepatitis before they are allowed to donate blood. One more critical point raised was that the monetary pay-out to the donors would increase the cost of acquiring blood transfusions and may make them out of the reach of certain individuals who cannot afford them. While we agree that this would increase the cost of acquiring blood and blood products, the scenario is not very different from how healthcare currently works in India. Pricing of products will ultimately be determined by market dynamics and supply-demand ratios. If supply is large and in surplus, prices are unlikely to significantly increase. Even if prices for blood products at certain blood banks increase, governments and non-profit organizations should continue to run blood banks in government facilities or as stand-alone blood banks, which can continue to function in the current model of not giving monetary compensation for blood donation, and therefore the cost of acquiring blood products would be the same as the prevailing rates. Direct blood donations, which constitute the majority of current blood donations, where a donor donates specifically for a known person, would not require this monetary compensation and, therefore, be unaffected. The monetary compensation would, therefore, be restricted only to donors who are donating without any specific recipient for whom it is intended.

Conclusions

As we presented in our detailed point-by-point analysis of the ethics and practicality of providing monetary incentives for blood donation, we believe that there is no reason significant enough not to allow monetary incentives. The benefits would outweigh the possible drawbacks. Hence, we believe that a change in this law and its modification to adapt to the current needs of society is warranted. The final roadblock for this change is not ethics or practicality, but rather the preparedness of a robust, efficient system that can ensure the correct execution of such policies and have the ability to prevent misuse. So it is not a question of ‘if’ the law should change, but a question of ‘when’ the law should change. And the answer to the question will be when we have the systems in place to ensure the law is not misused when it arrives.
